# A single-center retrospective study comparing safety and efficacy of endoscopic biliary stenting only vs. EBS plus nasobiliary drain for obstructive jaundice

**DOI:** 10.3389/fmed.2022.969225

**Published:** 2022-09-14

**Authors:** Huan Liu, Chuanke Shi, Zhideng Yan, Ming Luo

**Affiliations:** Department of General Surgery, Zhongshan Hospital of Traditional Chinese Medicine, Guangzhou University of Traditional Chinese Medicine, Zhongshan, China

**Keywords:** extrahepatic obstructive jaundice, endoscopic biliary drainage (EBD), endoscopic nasobiliary drainage (ENBD), endoscopic biliary stent (EBS), efficacy

## Abstract

**Purpose:**

Biliary drainage is an important modality for extrahepatic obstructive jaundice both in patients with palliative and resectable. Currently, endoscopic biliary drainage is preferred in clinical practice, including endoscopic nasobiliary drainage (ENBD) and endoscopic biliary stenting (EBS), both of which have their own advantages and disadvantages. The purpose of our study was to compare the safety and efficacy of endoscopic biliary stenting (EBS) only vs. EBS plus nasobiliary drain for obstructive jaundice.

**Methods:**

We consecutively reviewed patients with endoscopic biliary drainage in our institution from November 2014 to March 2021. Combined (ENBD plus stent) and single approach (EBS only) were defined as combined approach and single modality, respectively, and all eligible patients were divided into a combined approach group and a single modality group. We compared combined vs. single modality approaches to investigate whether there were statistical differences in liver chemistries, postoperative adverse events, and stent patency time.

**Results:**

In 271 patients, a total of 356 times endoscopic biliary drainages were performed. All eligible patients were divided into the combined approach group (*n* = 74) and the single modality group (*n* = 271). The combined approach was associated with a lower incidence of postoperative cholangitis and bleeding and greater improvement in liver chemistries, although it was not statistically significant. However, it was superior to the single modality group in terms of hospital stay (12.7 ± 5.2 vs. 14.5 ± 7.9 days, *p* = 0.020 < 0.05) and stent patency time (8.1 ± 3.9 vs. 4.3±2.7 months, *p* = 0.001 < 0.05).

**Conclusion:**

Endoscopic combined (ENBD plus stent) drainage is a more advantageous biliary drainage method that is characterized by more adequate biliary drainage, a lower incidence of postoperative adverse events, and longer effective biliary drainage time.

## Introduction

Jaundice is defined as serum bilirubin ≥2 mg/dl, of which obstructive jaundice is the most common in the surgical department. It is referred to as surgical jaundice because it requires surgical intervention due to the blockage of the intrahepatic and extrahepatic bile ducts. However, obstructive jaundice is almost caused by the obstruction of extrahepatic bile ducts, such as bile duct stones, benign strictures, metastatic carcinomas, bile duct, pancreatic, and duodenal tumors. Endoscopic retrograde cholangiopancreatography (ERCP) is an important modality for the treatment of obstructive jaundice due to its superiority and availability. Improved diagnostic imaging and surgical procedures have clear benefits for the management of obstructive jaundice, however, we know that most malignant obstructive jaundice has lost the opportunity for radical surgery when it is identified. Biliary drainage has become the most important palliative treatment for these patients, which not only improves their quality of life (QOL), such as relieving jaundice and severe pruritus but also improves their survival rate (1, 2). Similarly, preoperative biliary drainage (PBD) is an essential procedure for resectable patients, which not only improves liver chemistries, coagulation, and nutritional status, but also improves immune function, promotes liver regeneration, and reduces the risk of intraoperative and postoperative complications (3–6). In addition, another study confirmed that PBD can improve postoperative mortality, morbidity, and resection rate (7).

Current biliary drainage includes percutaneous transhepatic biliary drainage (PTCD), endoscopic nasobiliary drainage (ENBD), and endoscopic biliary stenting (EBS), all of which are used clinically due to their own advantages and disadvantages. PTCD is still the main procedure for alleviating jaundice in institutions without ERCP-related equipment and professionally trained endoscopists. However, it is a more invasive procedure, which not only has the risk of adverse events such as cholangitis, pancreatitis, bleeding, and liver abscess, but also has the risk of tumor implantation (8, 9). Considering the patient's quality of life and avoiding tumor spread and serious complications, surgeons prefer endoscopic biliary drainage (10–12), which includes ENBD and EBS. Unlike ENBD, which not only allows us to observe biliary drainage more directly, regularly flush and dredge the nasobiliary duct and perform cholangiography, but also allows for cytology (13) and microbial culture to guide subsequent treatment, EBS does not. EBS has a higher incidence of cholangitis than ENBD due to stent obstruction and intestinal bacterial reflux (14–17). However, it has advantages in liver chemistries and immune function by maintaining intestinal hepatic circulation, metabolism, and vitamin absorption (18, 19). It also has the advantage of being aesthetically pleasing and free of nasopharyngeal discomfort. Plastic and nasobiliary ducts are common in developing countries due to the expense and availability of metal stents. At present, there is no consensus on the choice of endoscopic biliary drainage, which is usually based on the clinical experience of the institution and patient preferences.

Therefore, we hypothesized that endoscopic combined (ENBD plus stent) drainage is superior to EBS alone for obstructive jaundice. The purpose of our study was to compare the safety and efficacy of endoscopic biliary stenting (EBS) only vs. EBS plus nasobiliary drain for obstructive jaundice.

## Materials and methods

### Patients

Our study is a single-center retrospective cohort study, which was reviewed by the Ethics Committee of Zhongshan Hospital of Traditional Chinese Medicine, Guangzhou University of Traditional Chinese Medicine and waived the ethical requirements. All patients obtained written informed consent. We consecutively reviewed patients with obstructive jaundice who underwent endoscopic biliary drainage in our institution between November 2014 and March 2021. All data were obtained through electronic medical record systems and telephone follow-ups. The inclusion and exclusion criteria for this study are as follows. Inclusion criteria: (a) total serum bilirubin (TSB) > 2 mg/dl; (b) obstructive jaundice identified by computed tomography (CT), magnetic resonance cholangiopancreatography (MRCP) or ERCP; and (c) cause of biliary obstruction determined by imaging or endoscopic pathology. Exclusion criteria: (a) patients with intrahepatic biliary obstruction; (b) patients with other causes of jaundice, such as hepatocellular and hemolytic jaundice; (c) patients with missing primary data; and (d) patients with only ENBD.

Data collected included clinical characteristics, ERCP procedures, and their efficacy. Clinical characteristics included gender, age, etiology of obstructive jaundice, number of ERCPs, diabetes mellitus, history of malignancy and surgery, and preoperative cholangitis and pancreatitis. The ERCP procedures included whether or not to perform endoscopic sphincterotomy (EST), method of biliary drainage, type and number of stents, operation time, and technical success rate. The efficacy indicators included length of hospital stay, liver chemistries, postoperative cholangitis, pancreatitis and bleeding, and stent patency time. Liver chemistries indicators included serum alkaline phosphatase (ALP), glutamyl transpeptidase (GGT), serum total bilirubin (TSB), alanine aminotransferase (ALT), and aspartate aminotransferase (AST).

The diagnosis of cholangitis is based on clinical manifestations, such as fever, abdominal pain, jaundice, shock, altered consciousness, and increased white blood cells (WBCs) and serum total bilirubin. The diagnosis of pancreatitis is based on a patient's serum amylase level >3 times the upper limit of normal, or clinical manifestations, such as fever and abdominal pain, and imaging studies, such as abdominal ultrasound, CT, or magnetic resonance imaging (MRI). Postoperative bleeding was defined as symptoms, such as melena, hematemesis, or ENBD with bloody drainage, or a decrease in hemoglobin of ≥20 g/L requiring blood transfusion.

Combined (ENBD plus stent) and stent only were defined as combined approach and single modality, respectively, and all eligible patients were divided into the combined approach group (*n* = 74) and the single modality group (*n* = 271).

### Endoscopic procedures

All ERCP procedures were performed by surgeons who perform more than 200 ERCPs per year. Both groups used the same endoscopic treatment system (such as, duodenoscope, contrast agent, contrast method, and pressure), the same anesthesia method, and postoperative follow-up. The only difference between the two groups was that the combined approach group had ENBD. All placed ENBDs were the same length and were sized to match the bile duct diameter and metal stent.

### Statistical analysis

Categorical variables and normally distributed quantitative variables were represented by frequency (percentage) and mean ± standard deviation (SD), respectively. Based on the characteristics of the data, we appropriately applied the *t*-test, the chi-square test, and Fisher's exact test to assess differences between groups. While the Wilcoxon and Kruskal–Wallis tests were applied for non-normally distributed data. All statistical analysis were performed with SPSS version 25 (IBM, Armonk, NY, USA). A two-sided *p* < 0.05 was used to indicate statistical significance. All methods in our studies were carried out in accordance with relevant guidelines and regulations.

## Results

**Figure 1:**
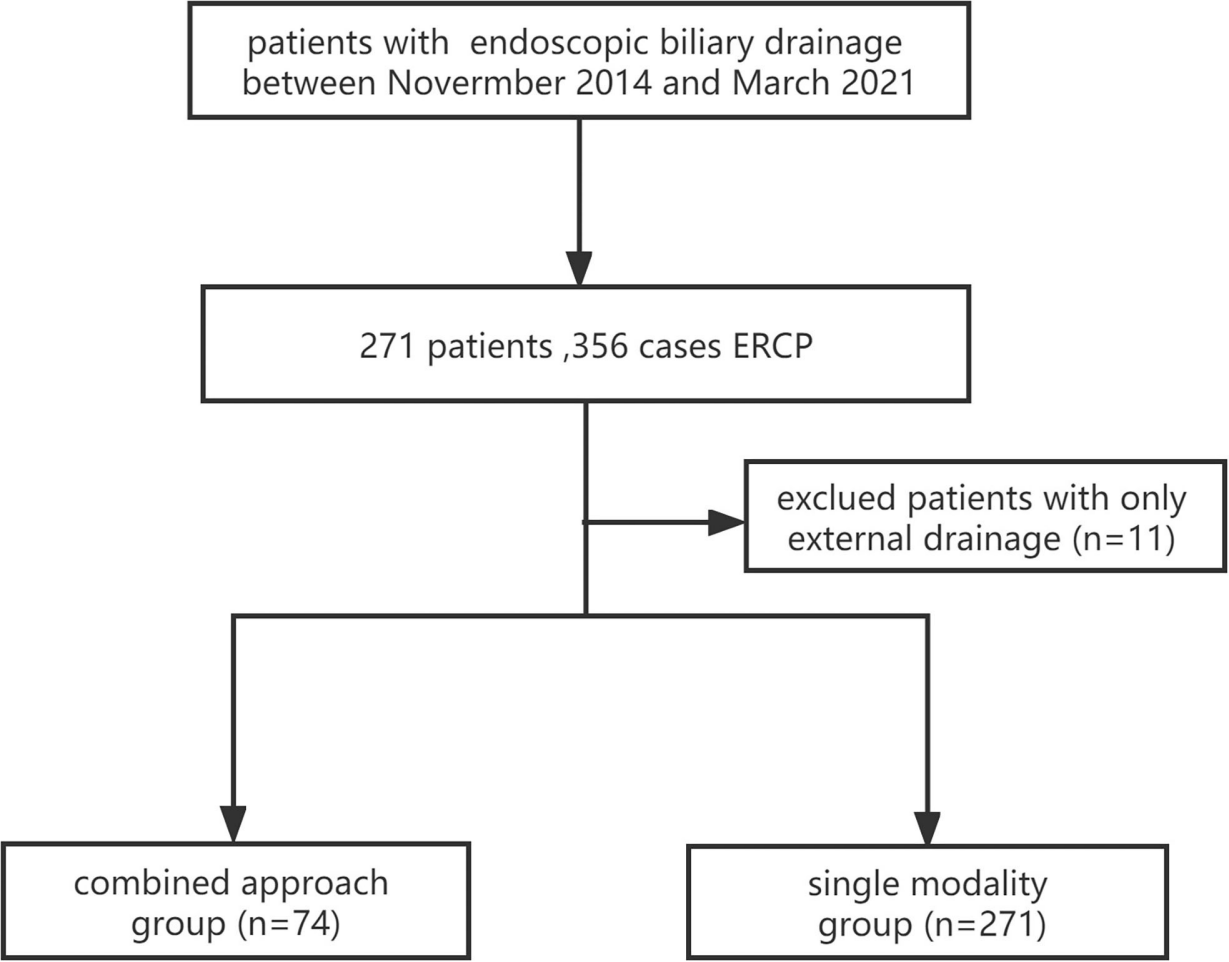
A flowchart of the study (the combined approach group: ENBD plus stent; and the single modality group: stent only).

The flow of the study is shown in Figure 1.

A total of 271 patients with obstructive jaundice underwent endoscopic biliary drainage from November 2014 to March 2021, with a total of 356 ERCPs. Among these patients, 11 patients with ENBD only were excluded, and the eligible patients were divided into the single modality group (*n* = 271) and the combined approach group (*n* = 74).

The clinical characteristics of eligible patients are shown in Table 1. The mean age of all eligible patients was 67.8 ± 11.2 years, and there were 202 (58.6%) men and 143 (41.4%) women, respectively. The causes of obstructive jaundice were extrahepatic cholangiocarcinoma, pancreatic cancer, ampullary cancer, large common biliary stones, metastatic lesions, and others. The other was a patient with sclerosing cholangitis. The primary tumors of 6 metastatic cancers were two liver cancer, two gallbladder cancer, one gastric cancer, and one colon cancer.

**Table 1:** Clinical characteristics of eligible patients.

**Characteristics**	
Age, y (mean ± SD)	67.8 ± 11.2
**Sex**, ***n*** **(%)**	
Male	202 (58.6)
Female	143 (41.4)
**Causes of extrahepatic biliary obstruction**, ***n*** **(%)**	
Cholangiocarcinoma	
Hilar	64 (18.6)
Non-hilar	86 (24.9)
Pancreatic cancer	94 (27.2)
Ampullary carcinoma	37 (10.7)
Large CBD stones	31 (9.0)
Malignant tumor metastasis	6 (1.7)
Inflammatory stricture	
With common biliary stones	12 (3.5)
Without common biliary stones	14 (4.1)
Other	1 (0.3)

SD, standard deviation; CBD, common bile duct.

The clinical characteristics of the two groups are shown in Table 2. There were no statistically significant differences between the two groups in mean age, sex, diabetes, preoperative cholangitis and pancreatitis, and history of cholecystectomy and malignancy. The etiology of obstruction was statistically significant between the two groups (*p* = 0.001), and there were more patients with bile duct stones and inflammatory strictures in the single modality group than in the combined group. We saw that the proportion of previous ERCPs in the single modality group was significantly higher than that in the combined approach group (37.6 vs. 13.5%, *p* = 0.001), especially the number of ERCPs ≥ 2. Although the proportion of liver metastases and distant metastases in the combined approach group was higher than that in the single modality group, there was no statistical difference (8.1 vs. 6.6%, *p* = 0.660; 16.2 vs. 9.3%, *p* = 0.051).

**Table 2:** Clinical characteristics of the two groups.

**Characteristics**	**Combined approach group (*n* = 74)**	**Single modality group (*n* = 271)**	***P*-value**
Age, y (mean ± SD)	69.2 ± 10.4	67.4 ± 11.4	0.206
**Sex**, ***n*** **(%)**			0.051
Male	36 (48.7)	166 (61.3)	
Female	38 (51.3)	36 (38.7)	
Causes of extrahepatic biliary obstruction			0.001
**Cholangiocarcinoma**			
Hilar	15 (20.3)	49 (18.1)	
Non-hilar	20 (27.0)	66 (23.4)	
Pancreatic cancer	32 (43.2)	62 (22.9)	
Ampullary carcinoma	6 (8.1)	31 (11.4)	
Large CBD stones	0 (0.0)	31 (11.4)	
Malignant tumor metastasis	1 (1.4)	5 (1.8)	
Inflammatory stricture			
With common biliary stones	0 (0.0)	14 (5.2)	
Without common biliary stones	0 (0.0)	12 (4.4)	
Other	0 (0.0)	1 (1.4)	
**ERCP** ***n*** **(%)**			0.001
Yes	10 (13.5)	102 (37.6)	
No	64 (86.5)	169 (62.4)	
**ERCP number** ***n*** **(%)**			
0	64 (86.5)	169 (62.4)	
1	8 (10.7)	61 (22.5)	
2	0 (0.0)	26 (9.6)	
3	1 (1.4)	10 (3.7)	
≥4	1 (1.4)	5 (1.8)	
**Diabetes** ***n*** **(%)**			0.792
Yes	7 (9.5)	23 (8.5)	
No	67 (90.5)	248 (91.5)	
**Previous cholecystectomy** ***n*** **(%)**			0.105
Yes	2 (2.7)	22 (8.1)	
No	72 (97.3)	249 (91.9)	
**History of malignant tumor** ***n*** **(%)**			0.220
Yes	6 (8.1)	11 (4.1)	
No	6 (91.9)	260 (95.9)	
**Liver metastases on admission** ***n*** **(%)**			0.660
Yes	6 (8.1)	18 (6.6)	
No	68 (91.9)	253 (93.4)	
**Distant metastasis on admission** ***n*** **(%)**			0.051
Yes	12 (16.2)	23 (9.3)	
No	62 (83.8)	248 (90.7)	
**Preoperative pancreatitis** ***n*** **(%)**			1.000
Yes	1 (1.4)	5 (1.8)	
No	73 (98.6)	266 (98.2)	
**Preoperative cholangitis** ***n*** **(%)**			0.105
Yes	2 (2.7)	22 (8.1)	
No	72 (97.3)	249 (91.9)	

SD, standard deviation; CBD, common bile duct.

The ERCP procedures in the two groups are shown in Table 3. During ERCP, EST is usually performed to facilitate stent implantation and removal of common biliary stones. We saw that the proportion of EST was higher in the combined approach group than in the single modality group (72.9 vs. 31%, *p* = 0.001). There were 2 patients with failed stent placement in the single modality group, but none in the combined approach group. However, there was no significant difference in the technical success rate between the two groups (99.3 vs. 100%, *p* = 1.000). To prevent or relieve pancreatitis, we usually place pancreatic plastic stents in selected patients. We saw no difference in the proportion of pancreatic duct stents between the two groups (14.9 vs. 14.0%, *p* = 0.854). We saw that the proportion of multiple biliary stents placed in the single modality group was significantly higher than that in the combined approach group (36.5 vs. 20.3%, *p* = 0.008). The single modality group preferred multiple plastic stents, however, the combined approach group was mostly a single metal stent.

**Table 3:** Comparison of the combined approach group and the single modality group in ERCP procedures.

**Characteristics**	**Combined approach group**	**Single modality group**	***P*-value**
**EST** ***n*** **(%)**			0.001
Yes	54 (72.9)	84 (31.0)	
No	20 (27.1)	187 (69.0)	
**EBS technical success rate** ***n*** **(%)**			1.000
Yes	74 (100.0)	269 (99.3)	
No	0 (0.0)	2 (0.7)	
**Pancreas stenting** ***n*** **(%)**			0.854
Yes	11 (14.9)	38 (14.0)	
No	63 (85.1)	233 (86.0)	
**Number of biliary stents** ***n*** **(%)**			0.008
1	59 (79.7)	172 (63.5)	
≥2	15 (20.3)	99 (36.5)	
**Number and types of biliary stents** ***n*** **(%)**			
*N* = 1	59 (79.7)	173 (63.8)	
Metal	57 (96.6)	64 (37.2)	0.001
Plastic	2 (3.4)	109 (62.8)	
*N* = 2	15 (20.3)	80 (29.5)	
1Metal + 1Plastic	5 (33.3)	12 (15.0)	0.135
2Plastic	10 (66.7)	68 (85.0)	
*N* ≥ 3	0 (0)	17 (6.3)	
1Metal + 2Plastic	0 (0.0)	6 (35.3)	
3Plastic	0 (0.0)	10 (58.8)	
4Plastic	0 (0.0)	1 (5.9)	

ERCP, endoscopic retrograde cholangiopancreatography; EST, endoscopic sphincterotomy; EBS, endoscopic biliary stenting.

The laboratory parameters are shown in Table 4. In terms of preoperative-postoperative changes in WBC, it was decreased in both groups, and the difference was statistically significant in the single modality group, but not in the combined approach group. However, its change was not statistically different between the two groups (0.4 ± 4.2 vs. 0.6 ± 3.8, *p* = 0.719). We saw a statistically significant decrease in hemoglobin (HB) in the combined approach group and the single modality group (11.2 ± 14.1, *p* = 0.001, 7.9 ± 15.2, *p* = 0.001), however, there was no difference in the change in HB (11.2 ± 14.1 vs. 7.9 ± 15.2, *p* = 0.107). After ERCP, ALP decreased statistically in the two groups (638.5 ± 396.6 vs. 436.2 ± 335.0 U/L, *p* = 0.001 and 649.8 ± 385.5 vs. 433.3 ± 245.1 U/L, *p* = 0.001). Although there was no difference in ALP change between the two groups, the combined approach group was more significant than the single modality group (182.2 ± 191.4 vs. 142.6±233.9 U/L, *p* = 0.185). There were statistically significant differences in GGT changes between the combined approach group and the single modality group (671.1 ± 596.9 vs. 321.1 ± 225.5 U/L, *p* = 0.001 and 514.2 ± 386.0 vs. 329.9 ± 223.4 U/L, *p* = 0.001), and the change was more significant in the combined approach group (351.1 ± 492.2 vs. 184.7 ± 345.6 U/L, *p* = 0.008). TSB decreased statistically in the two groups (212.1 ± 138.3 vs. 98.3 ± 79.8 μmol/L, *p* = 0.001 and 182.4 ± 178.3 vs. 84.4 ± 80.1 μmol/L, *p* = 0.001). The change was more significant in the combined approach group than in the single modality group (113.8 ± 108.3 vs. 98.5 ± 153.9 μmol/L, *p* = 0.425). We saw that in the two groups, liver chemistries had been significantly improved, and there was no difference in the changes between the two groups. The changes in ALT and AST were 90.5 ± 100.7 U/L (*p* = 0.001), 64.1 ± 82.6 U/L (*p* = 0.001) in the combined approach group, and 86.2 ± 132.8 U/L, 76.5 ± 107.3 U/L (*p* = 0.001) in the single modality group. There was no significant difference in ALT and AST changes between the two groups (*p* = 0.803 and *p* = 0.370).

**Table 4:** Laboratory parameters in the combined approach group and the single modality group.

	**Combined approach group**			**Single modality group**			
	**Pre-ERCP**	**D**	***P*-value**	**Pre-ERCP**	**D**	***P*-value**	*P*-value*
WBC (×109/L)	7.3 ± 3.0	0.4 ± 4.2	0.465	7.4 ± 3.6	0.6 ± 3.8	0.019	0.719
HB (×1,012/L)	118.1 ± 19.4	11.2 ± 14.1	0.001	117.3 ± 20.4	7.9 ± 15.2	0.001	0.107
ALP (U/L)	638.5 ± 396.6	182.2 ± 191.4	0.001	649.8 ± 385.5	142.6 ± 233.9	0.001	0.185
GGT (U/L)	671.1 ± 596.9	351.1 ± 492.2	0.001	514.2 ± 386.0	184.7 ± 345.6	0.001	0.008
TSB (μmol/L)	212.1 ± 138.3	113.8 ± 108.3	0.001	182.4 ± 178.3	98.5 ± 153.9	0.001	0.425
ALT (U/L)	143.8 ± 113.5	90.5 ± 100.7	0.001	141.8 ± 145.4	86.2 ± 132.8	0.001	0.803
AST (U/L)	118.0 ± 83.9	64.1 ± 82.6	0.001	124.4 ± 109.6	76.5 ± 107.3	0.001	0.370

The p-value^*^: D (combined approach) vs. D (single modality group).

D, pre-ERCP minus post-ERCP.

ALP, alkaline phosphatase; ALT, alanine aminotransferase; AST, aspartate aminotransferase; GGT, γ-glutamyl transpeptidase; TSB, total serum bilirubin; WBC, white blood cell; HB, hemoglobin; SD, standard deviation; post-ERCP. The post-ERCP indicators are not shown, but can be provided if needed.

The efficacy and adverse events after EBS are shown in Table 5. The length of hospital stay was significantly shorter in the combined approach than in the single modality (12.7 ± 5.2 vs 14.5 ± 7.9 days, *p* = 0.020). We found a higher incidence of postoperative pancreatitis in the combined approach group than in the single modality group (16.2 vs. 10.2%), however, the results were reversed for postoperative cholangitis and bleeding (2.7 vs. 8.1 and 2.7 vs. 6.3%), but these were not statistically significant. In terms of stent patency time, we obtained detailed data on stent patency time, with 14 patients in the combined approach group and 56 patients in the single modality group. The shortest and longest times were 3 and 18 months in the combined approach group, respectively, and 15 days and 16 months in the single modality group. We saw that the stent patency time in the combined approach group was significantly longer than that in the single modality group (8.1 ± 3.9 vs. 4.3 ± 2.7 months, *p* = 0.001).

**Table 5:** Comparison of postoperative adverse events and efficacy in the two groups.

	**Combined approach group**	**Single modality group**	***P*-value**
Length of hospital stay (day)	12.7 ± 5.2	14.5 ± 7.9	0.020
Postoperative pancreatitis *n* (%)			0.161
Yes	12 (16.2)	28 (10.3)	
No	62 (83.8)	243 (89.7)	
Postoperative cholangitis *n* (%)			0.105
Yes	2 (2.7)	22 (8.1)	
No	72 (97.3)	249 (91.9)	
Postoperative bleeding *n* (%)			0.387
Yes	2 (2.7)	17 (6.3)	
No	72 (97.3)	254 (93.7)	
Stent patency time (month)			
N*	14	56	0.001
(mean ± SD)	8.1 ± 3.9	4.3 ± 2.7	

SD, standard deviation.

N^*^, the number of patients whose stent patent time is available.

## Discussion

We know that the most common causes of extrahepatic obstructive jaundice are cholangiocarcinoma, pancreatic cancer, and ampullary malignancies. When the disease is recognized, they are inoperable due to the advanced stage. Therefore, biliary drainage to relieve hyperbilirubinemia has become the most important treatment for these patients (20, 21). It not only relieves severe itching to improve the quality of life, but also restores the enterohepatic circulation of bilirubin to improve the nutritional status of patients, and alleviates the effects of hyperbilirubinemia on other organ functions (22), which are beneficial to the survival of patients. In addition, PBD is an essential procedure for those patients who can be radically resected (19), which reduces postoperative adverse events and improves survival. Some studies have shown that the median survival time of cholangiocarcinoma patients with bilirubin > 2 mg/dl was 4.8 months (95% *CI*, 3.1–9.4 months), however, 15.2 months (95% *CI*, 11.7–19.3 months) in patients with bilirubin ≤2 mg/dl (23). Similarly, another study also confirmed that even in patients with normal bilirubin, successful biliary drainage improves patient survival (1). In the institutions available for ERCP, the current biliary drainage prefers endoscopic biliary drainage over PTCD. Infection after ERCP is an inevitable serious complication, and its fatality rate is 8–20% (24, 25), and EBS increases its risk even more. Although EBS unblocks the passage of bile from the liver or gallbladder into the gut, it disrupts the mechanical and functional anti-reflux barriers, leading to an increased risk of acute cholangitis, pancreatitis, and liver abscesses, especially in the lower common biliary carcinoma and ampullary malignancies.

For patients with malignant obstructive jaundice, the median survival even after radical surgery is 1–4 years (26), whereas for patients with palliative biliary drainage may be only a few months. Therefore, the choice of the biliary drainage method needs to take into account the improvement of liver chemistry, postoperative adverse events, and effective drainage time. Our study found that the combined approach was superior to the single modality in terms of the stent patency time (8.1 ± 3.9 vs. 4.3 ± 2.7 months, *p* = 0.001). Repeated hospitalizations and multiple ERCPs not only increase the economic and physical burden but also cause mental distress and affect the quality of life in the end stage. Longer stent patency times mean fewer stent replacements, and the clinical characteristics of patients provide some evidence to some extent. In our study, we saw that the proportion of previous ERCPs was significantly higher in the single modality group than in the combined approach group (37.6 vs. 13.5%, *p* = 0.001), and even more so in multiple ERCPs. Our ideal biliary drainage would be better-improved liver chemistry, lower postoperative adverse events, and longer functional biliary drainage. In terms of improvement in liver chemistry indications in our study, we saw significant improvements in ALP, GGT, TSB, ALT, and AST in both groups, and more significantly in the combined approach group, but it was not statistically significant. Similarly, the incidence of postoperative cholangitis and bleeding in the combined approach group was lower than that in the single modality group, and the difference was not statistically significant. However, considering the differences in liver and distant metastases between the two groups at admission (8.1 vs. 6.6%, *p* = 0.660 and 16.2 vs. 9.3%, *p* = 0.051), we can see that the condition was more serious in the combined approach group. Therefore, this provided some evidence to some extent that combined (ENBD plus stent) drainage is more advantageous than stent drainage only in extrahepatic biliary obstructive jaundice.

In our study, we saw that the single modality group was more inclined to place multiple biliary stents than the combined approach group (36.5 vs. 20.3%, *p* = 0.008). Moreover, we found that the single modality group tended to be more plastic (62.8 vs. 3.4%, *p* = 0.001), even when placing multiple stents, there would be more choices for placing multiple metal stents. The cost of a single plastic stent is slightly lower than that of a metal stent. However, in clinical practice, surgeons usually tend to place multiple plastic stents for adequate drainage, and the overall cost is not less than that of metal stents. Moreover, multiple previous studies suggested that patients with bilateral multiple biliary stent drainage had an increased incidence of adverse events, such as cholangitis and liver abscess after ERCP (27–29). According to our clinical experience, patients with multiple common biliary plastic stents complain of upper abdominal discomfort, especially in patients with ≥3 plastic stents. The study by Cassani et al. showed that in the drainage of hilar biliary malignant tumors, there was no significant difference between plastic stents and metal stents in clinical success rate, cholangitis, acute pancreatitis, and stent displacement. However, in terms of stent blockage and tumor growth, plastic stents are obviously superior to metal stents (23). However, in current clinical practice, metal stents are almost fully covered with self-expanding metal stents, and compared with plastic stents, there is no significant difference in tumor growth in the stent. Moreover, when the metal stent is blocked, we need to unclog or replace it with ERCP again. However, the plastic stent needs to be replaced. Multiple studies have confirmed that fully covered metal stents have longer patient survival, a lower risk of stent dysfunction, cholangitis, and fewer re-interventions (3, 20, 30–32), and the patient's health-related quality of life (such as, general and disease-specific) is better (33). The study by Zhang et al. showed that patients with ENBD for biliary drainage had a significantly lower incidence of acute cholangitis, pancreatitis, and stent dysfunction (34).

Therefore, we prefer endoscopic combined (ENBD plus stent) drainage for patients with extrahepatic obstructive jaundice, especially for patients with malignant tumors of the lower end of the common biliary and ampulla. However, multiple studies have shown that ENBD is lower than EBS in postoperative cholangitis (14, 16, 35, 36) and stent dysfunction (14, 18, 19, 36) for malignant biliary obstruction drainage. A previous study showed that the temporary placement of ENBD in patients with fully covered self-expanding metal stents can reduce the incidence of postoperative cholangitis (37). Similarly, the bridge preoperative biliary drainage, that is, ENBD is replaced with a biliary stent when it is dysfunctional or intolerant, which can shorten the preoperative hospital stay and have a longer preoperative biliary drainage time (38). In our study, combined (ENBD plus stent) drainage can adequately drain the biliary tract and reduce the number of stents and procedure time, which fully utilizes ENBD and EBS. It is manifested as a more advantageous biliary drainage method, with sufficient biliary drainage, a lower incidence of postoperative adverse events, and longer successful biliary drainage time. We know that patients with malignant biliary obstruction are mostly elderly and have multiple underlying diseases. The longer the procedure time, the higher the incidence of postoperative adverse events, which affect the morbidity and mortality of patients. The causes of biliary stent dysfunction are usually refluxed food particles, blood clots, sludge, and small stones. For patients with ENBD plus stent drainage, when the biliary drainage dysfunction occurs, we can remove the ENBD or unblock the biliary stent, and the biliary drainage may be successful again.

Although, nasobiliary drainage is undoubtedly a good option for drainage, its displacement and nasopharyngeal discomfort are unavoidable problems, which lead to nasobiliary ducts rarely used in the West. However, nasobiliary application is more common in Asia because malignant patients are more concerned with clinical symptoms, such as jaundice and pruritus rather than nasopharyngeal discomfort. Due to the above reasons, we could not include nasopharyngeal discomfort in our study. The advantage of EBS is more stability and immobilization. However, for patients with PBD, EBS may not be a good choice because of stent removal and the impact on subsequent surgery. The EBSplus stent in our study may be more suitable for those patients with unresectable malignant obstructive jaundice, which may prolong stent patency, reduce adverse events, and improve quality of life. Compared with EBS alone, it is also possible to provide patients with psychological comfort and support, which is very important for patients with these advanced tumors, while its suitability for other patients remains to be studied.

This study also has many limitations. First, patient quality of life (QOL) is an important indicator, especially considering the limited life expectancy of patients with advanced malignancies, however, our study did not incorporate QOL measures. Second, our study assumes that the size of the biliary stent does not affect its function.

## Conclusion

In conclusion, combined (ENBD plus stent) drainage is a more advantageous biliary drainage method, which is characterized by more adequate biliary drainage, a lower incidence of postoperative adverse events, and a longer effective biliary drainage time.

## Data availability statement

The original contributions presented in the study are included in the article/supplementary materials, further inquiries can be directed to the corresponding author.

## Ethics statement

The studies involving human participants were reviewed and approved by the Ethics Committee of Zhongshan Hospital of Traditional Chinese Medicine, Guangzhou University of Traditional Chinese Medicine. The patients/participants provided their written informed consent to participate in this study.

## Author contributions

HL designed and performed the research and wrote the paper. ML designed the research and supervised the report. CS and ZY provided clinical advice. All authors read and approved the final manuscript. All authors contributed to the study conception and design. All authors read and approved the final manuscript.

## Conflict of interest

The authors declare that the research was conducted in the absence of any commercial or financial relationships that could be construed as a potential conflict of interest.

## Publisher's note

All claims expressed in this article are solely those of the authors and do not necessarily represent those of their affiliated organizations, or those of the publisher, the editors and the reviewers. Any product that may be evaluated in this article, or claim that may be made by its manufacturer, is not guaranteed or endorsed by the publisher.
